# Wnt5a Is Strongly Expressed at the Leading Edge in Non-Melanoma Skin Cancer, Forming Active Gradients, while Canonical Wnt Signalling Is Repressed

**DOI:** 10.1371/journal.pone.0031827

**Published:** 2012-02-22

**Authors:** Celine Pourreyron, Louise Reilly, Charlotte Proby, Andrey Panteleyev, Colin Fleming, Kathleen McLean, Andrew P. South, John Foerster

**Affiliations:** 1 Medical Research Institute, College of Medicine, Dentistry, and Nursing, University of Dundee, Dundee, Scotland; 2 Department of Dermatology, College of Medicine, Dentistry, and Nursing, University of Dundee, Dundee, Scotland; 3 Cancer Research UK Cancer Centre Dundee, College of Medicine, Dentistry, and Nursing, University of Dundee, Dundee, Scotland; 4 Education Division, College of Medicine, Dentistry, and Nursing, University of Dundee, Dundee, Scotland; 5 Tayside Tissue Bank, College of Medicine, Dentistry, and Nursing, University of Dundee, Dundee, Scotland; Ohio State University Medical Center, United States of America

## Abstract

Wnt5a is one of the so-called non-canonical Wnt ligands which do not act through β-catenin. In normal development, Wnt5a is secreted and directs the migration of target cells along concentration gradients. The effect of Wnt5a on target cells is regulated by many factors, including the expression level of inhibitors and receptors. Dysregulated Wnt5a signalling facilitates invasion of multiple tumor types into adjacent tissue. However, the expression and distribution of Wnt5a in cutaneous squamous cell carcinoma (SCC) and basal cell carcinoma (BCC), as well as the effect of Wnt5a on keratinocyte migration has not been studied in detail to date. We here report that Wnt5a is upregulated in SCC and BCC and localised to the leading edge of tumors, as well as tumor-associated fibroblasts. The Wnt5a-triggered bundling of its receptor Fzd3 provides evidence of Wnt5a concentration gradients projecting into the tumor. In vitro migration assays show that Wnt5a concentration gradients determine its effect on keratinoctye migration: While chemotactic migration is inhibited by Wnt5a present in homogenous concentrations, it is enhanced in the presence of a Wnt5a gradient. Expression profiling of the Wnt pathway shows that the upregulation of Wnt5a in SCC is coupled to repression of canonical Wnt signalling. This is confirmed by immunohistochemistry showing lack of nuclear β-catenin, as well as absent accumulation of Axin2. Since both types of Wnt signalling act mutually antogonistically at multiple levels, the concurrent repression of canonical Wnt signalling suggests hyper-active Wnt5a signal transduction. Significantly, this combination of gene dysregulation is not observed in the benign hyperproliferative inflammatory skin disease psoriasis. Collectively, our data strongly suggest that Wnt5a signalling contributes to tissue invasion by non-melanoma skin cancer.

## Introduction

Wingless-type (Wnt) ligands are signalling molecules important in development. Wnt ligands are classified as “canonical” or “non-canonical” [1]. Canonical Wnts, exemplified by Wnt3a, bind to Fzd-type receptors, as well as LRP5/6 co-receptors, followed by the recruitment of a heteromeric protein complex including Dishevelled, Axin, and GSK3β to the receptor complex. This leads to phosphorylation of LRP5/6, release and nuclear translocation of β-catenin, culminating in the induction of target genes. By contrast, non-canonical Wnts, including Wnt5a, bind Fzd receptors in conjunction with alternate co-receptors, including ROR1/2 or Ryk, causing β-catenin-independent changes such as PKC activation and cytoskeletal rearrangements [2]. Importantly, by binding to common Fzd receptors, canonical and non-canonical Wnts act as competitive antagonists at shared receptors [3].

In development, secretion of all Wnt ligands including Wnt5a is subject to precise temporal and spatial control whereby concentration gradients are achieved [4]. These gradients direct morphogenetic movement of target cells as well as the arrangement of asymetrical polarisation of epithelial cells [5]. Thus, Wnt5a essentially directs migration of cells into surrounding tissue, for example in limb development. One key element determining the effect of Wnt on target cells is the presence of secreted inhibitory proteins. These include the Dickkopf (Dkk) family, which specifically bind LRP5/6, thus serving as specific inhibitors of canonical Wnts. Other inhibitors include Wif and the Secreted Frizzled Related Proteins (SFRP) which bind both types of Wnt ligands as well as Fzd receptors, thereby inhibiting both canonical and canonical Wnts [6]. The spatial distribution of SFRP, Fzd, Dkk, and Wnt is minutely orchestrated in development (e.g. [7], effectively creating diffusion corridors for Wnt activity.

Not surprisingly given its role as regulator of cell migration into adjacent tissue, the unregulated activation of Wnt5a has been associated with invasiveness and in several tumor types, including melanoma [8], [9], breast cancer [10], gastric cancer [11], pancreatic cancer [12], and osteosarcoma [13]. Wnt5a-related tumor invasion may also be mediated by tumor-associated cells. Thus, breast cancer cells induce Wnt5a expression in tumor-infiltrating macrophages, causing synthesis of matrix metalloproteinase (MMP) 7 [10].

Wnt5a can bind several frizzled receptors, including Fzd2, Fzd5, Fzd3, Fzd4. Of these, we have previously shown that Fzd5 and Fzd3 are expressed in the parental tissue for both squamous cell carcinoma (SCC), the epidermis, and basal cell carcinoma (BCC), the hair follicle, respectively [14]. These Fzd receptor isoforms have also been shown to mediate Wnt5a-induced directional motility in melanoma [15], as well as invasive migration in breast cancer [16]. Importantly, Fzd3 has recently been shown to accumulate into polarised focal aggregates when cells are exposed to a Wnt5a gradient in vitro [15]. While Wnt5a gradients cannot be detected directly in primary tissue, this discovery opens the possibility of utilising the intracellular distribution of Fzd3 as indicator of functional Wnt5a gradients acting on cells *in vitro*.

Non-melanoma skin cancer comprising BCC and SCC is the commonest human cancer and still increasing in incidence with more than 100,000 cases diagnosed each year in the UK. Although only SCC metastasizes in immunocompetent individuals, SCCs are highly locally invasive, making them readily available natural models to study tissue invasion. The expression of Wnt5a and cognate receptors has not been studied on the protein level in these tumors and few data exist on the effect of Wnt5a on directional migration in SCC cells or keratinocytes in vitro.

Here we report the distribution of Wnt5a and the receptors Fzd5 and Fzd3 in SCC and BCC. We utilise Fzd3 localisation to identify Wnt5a gradients operative in adult skin as well as in SCC/BCC. We further provide functional evidence that Wnt5a gradients enhance directed motility of keratinocytes. Expression profiling shows that invasive SCC, in contrast to benign inflammatory hyperproliferation of keratinocytes, is marked by the concurrent upregulation of non-canonical and repression of canonical Wnt signalling. Our data allow the formulation of a working model of how Wnt5a may act to enhance motility and invasiveness in these tumors.

## Materials and Methods

### Ethics statement

Prior to biopsy, patients gave written consent to storage and analysis of biopsy samples. Storage and use of all tissues included in the work presented here was carried out in accordance with the Helsinki declaration and approved by the Tayside Committee on Medical Research Ethics B (REC ref. Nr. 07/S1402/90).

### Tumor samples

SCC studied here were excised from immunocompetent patients from the head (n = 7) or the hands/legs (n = 4), in each case exhibiting surrounding signs of sun damage and classified as well-differentiated (n = 8), or moderately/poorly differentiated (n = 3). BCC (n = 9) were all from the head, except for one BCC excised from the hand.

### Immunohistochemistry

Antibodies used were anti Wnt5a (R&D, order nr. AF645, final dilution 1∶400, alternative antibody (shown in fig. S1: Abcam, clone 3D10, order nr. Ab86720, used at 1∶10000 dilution), anti-frizzled 3 (Insight Biotech, ordered through Acris Antibodies, Germany, order nr. SP4568P, 1∶200), anti-frizzled 5 (Cambridge Bioscience, ARP41245_P050, 1∶800), β-catenin (Millipore, 05–665). Immunohistochemistry was carried out exactly as described [14] using Paraffin-embedded samples obtained from the Tayside Tissue bank.

### Stable expression of Wnt5a and cell culture

#### Establishment of Wnt5a-stably transfected HaCat cells

HaCat cells were obtained as described previously [17]. Cells were plated in 6-wells cell culture plates at a density of 5×10^4^ cells/well prior to lipofectamine transfection with either pcDNA3 WNT5A-HA construct (full length human recombinant Wnt5a with C-terminal HA tag) or a pcDNA3 empty vector. Cells were transfected with 1 µg of plasmid DNA complexed with 8 µl Lipofectamine 2000 Transfection Reagent and incubated with transfection complex for 4 hours. Supernatant was removed and DMEM+10% FCS containing 800 µg/ml G418 was added to each well. Cells were observed over the course of two weeks, with media changes every 2–3 days. Once colonies started to appear, cells were trypsinised and transferred to 25 cm^2^ cell culture flasks. Cultures were maintained in DMEM containing 10% FCS (non-heat inactivated) with 800 µg/ml G418. Continued Wnt5a expression was verified by Western blot using anti- Wnt5a (R&D, AF645, dilution 1∶1000).

### Transwell migration assay

A Transwell system that incorporated a polycarbonate filter membrane with a diameter of 6.5 mm and pore size of 8 µm (Corning, Sigma-Aldrich, Poole, UK) was used to assess the rate of cell migration. Mitomycin C-treated cells (1×10^5^) were suspended in 100 µl of 0.1% BSA DMEM with or without human/mouse recombinant Wnt5a (0.1 µg/ml) (R&D, Abingdon, UK, order nr. 645-WN) and seeded in the upper chamber of the Transwell insert. The lower chamber was filled with 600 µl of DMEM supplemented with 5% FBS. Migration of HaCat-pcDNA cells in the presence of a Wnt5a concentration gradient was performed as follows. Wnt5a-overexpressing or pcDNA HaCat cells were seeded in 24 w/plates 48 h before adding the Transwell inserts until reaching confluence. Immediately prior to adding the Transwell inserts containing HaCat-pcDNA cells (in 0.1%BSA containing DMEM) the media in the wells were replaced by fresh 5%FBS DMEM to remove any pre-secreted Wnt5a. Plates were left for 18 h at 37°C. Subsequently, cells in the filters were stained with 1% Borax and 1% methylene blue. The nonmigrating cells on the upper surface of the filter were removed with a cotton swab and the cells that migrated to the lower surface of the filter were lysed with a solution of 1% SDS and quantified by measuring absorbance at 630 nm using a microplate spectrophotometer.

### Scratch wound migration assay

Cells were grown to confluence in a 6-well plate in Hacat medium. Two hours before wounding, cells were treated with mitomycin C (10 µg/ml) to prevent proliferation. A wound was made by applying a 200 µl plastic pipette tip across the centre of the cell sheet. Cells were washed twice with PBS and incubated in DMEM supplemented with either 10% or 1% FCS.

### Expression profiling

The log 2 transformed processed array data set was obtained from [18]), inverse-log2 data calculated, and fold-changes between SCC and sun-exposed skin control calculated for each case (n = 12). Average fold-changes and t-tests were then calculated as described [19]. Psoriasis expression profile analysis was done as described [19].

## Results

### Wnt5a is strongly expressed in non-melanoma skin cancer

**Figure 1:**
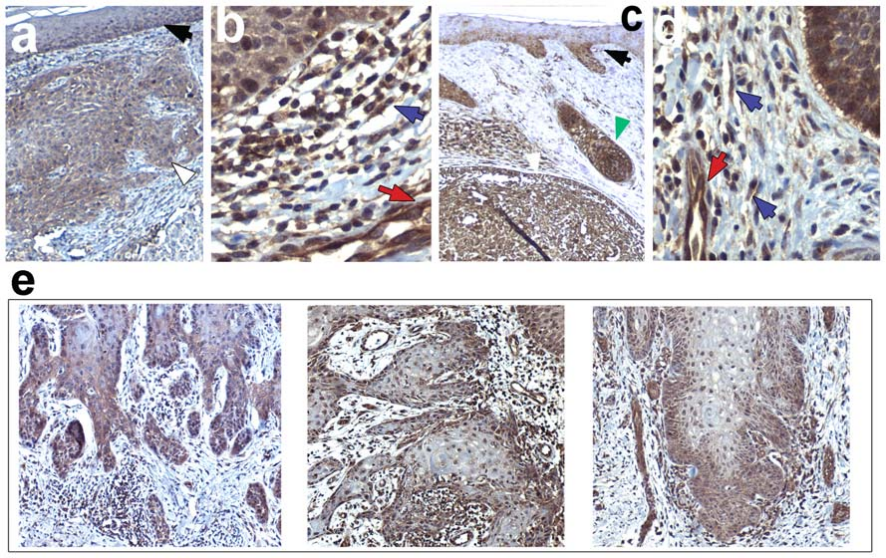
Localization of Wnt5a in non-melanoma skin cancer. Immunohistochemistry of Wnt5a from SCC (a,b), or BCC (c,d), shown at 40× (a,c), or 200× (b,d) magnification. (e) Three SCC tumors, shown at 10× magnification, illustrating strong Wnt5a – staining at the tumor edge. Figures shown are representative for SCC (n = 12), and BCC (n = 9), respectively. Arrowheads indicate the following structures: black - basal layer of the epidermis, white- tumors, red- tumor associated endothelial cells, blue- fibroblasts, green – hair follicle.

We studied Wnt5a expression in a panel of SCC (n = 11) and BCC (n = 9), excised from immunocompetent individuals, by immunohistochemistry. In order to allow semiquantitative assessment we used the previously characterised expression of Wnt5a in the basal layer or the epidermis [14] as internal calibration. As shown in figure 1, Wnt5a was strongly expressed in both SCC and BCC relative to its expression level in the basal layer of the epidermis (marked with black arrow) in the same sections (fig. 1a,c). Tumor-associated fibroblasts as well as endothelial cells also stained strongly positive for Wnt5a (fig. 1b,d, red and blue arrows, respectively). Although Wnt5a staining was detectable throughout tumors (example shown in fig. 1a), it was most intense at the leading edge of most tumors (fig. 1e). These findings were consistent between all studied tumor samples (table 1 and below) and were reproducible using an alternative antibody for IHC (figures S4, S5).

**Table 1:** Expression of Wnt5a, Fzd3, and Fzd5 in non-melanoma skin cancer.^1^

		Wnt5a	Frizzled 3	Frizzled 5
Level^2^	**SCC**	Moderate/strong	strong	Strong (9/12)weak/absent (3/12)
	**BCC**			Weak/absent (7/9)moderate (2/9)
Pattern	**SCC**	Highest at tumor edges	patchy (neg. vs. pos. zones)	patchy (neg. vs. pos.)
	**BCC**			
Intracellular distribution	**SCC**	homogenous	focal	homogenous
	**BCC**			
Associated fibroblasts	**SCC**	strong	partially positive	partially positive
	**BCC**			negative
Associatedvessels	**SCC**	strong	negative	strong
	**BCC**			

1 Immunohistochemistry of formaldehyde-fixed paraffin-embedded SCC (n = 12) and BCC (n = 9) samples was carried out as described in Methods.

2 Expression level was scored as “moderate” when staining intensity was comparable, as “strong” when staining was stronger, and as “low” when staining was weaker than that of epidermis present in the same section, respectively.

### Focal polarised distribution of Fzd3 in skin and non-melanoma skin cancer indicates Wnt5a gradients

**Figure 2:**
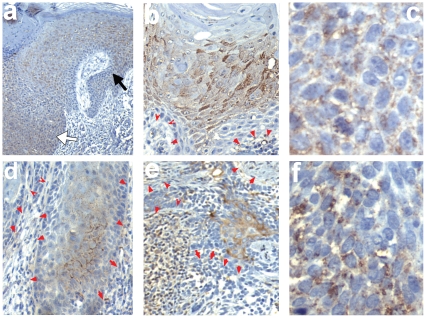
Localization of Fzd3 in non-melanoma skin cancer. Immunohistochemistry performed as in figure 1 performed on SCC (a–d), or BCC (e–f) shown at 40× (a), 100× (b,d,e), or 400× (c,f) magnification, respectively. Black arrows indicate staining intensity of Fzd3 in the epidermis used to assess staining intensity in tumors (denoted by white arrows). Red arrows denote boundary of tumors, pointing toward stroma.

Wnt5a concentration gradients cannot be directly detected in vivo. However, recently it was shown that, upon sensing a Wnt5a concentration gradient, target cells respond by bundling the Wnt5a receptor Fzd3 into focal aggregates in vitro [15]. Therefore, Fzd3 aggregates can be used as indirect marker to identify cells exposed to a Wnt5a gradients in primary tissue using immunohistochemistry. Indeed, we found that Fzd3 exhibited a strikingly polarised focal distribution both in epidermal keratinocytes as well as in the hair follicles (Fig. S1), suggesting that Wnt5a gradients are operative not only in development, but also in adult differentiated skin. Next, we investigated Fzd3 distribution in tumor sections. As with Wnt5a, we utilised the staining intensity of Fzd3 in the epidermis in each section to semiquantitatively assess the relative expression level (fig. 2a, black vs. white arrows). As shown in figure 2, Fzd3 was found in a zonal distribution, such that Fzd3-negative tumor areas alternate with Fzd3-positive areas (fig. 2b,e) within the tumors, while the invasive edges did not stain positive (fig. 2d). Tumor-infiltrating fibroblasts and endothelial cells were negative for Fzd3. In those tumor cells that did express Fzd3, Fzd3 exhibited a pronounced polarised focal intracellular aggregates, suggesting the existence of Wnt5a gradients. However, in contrast to normal epidermis, Fzd3-aggregates were not aligned along recogniseable planes, indicative of disorganised Wnt5a gradients. This overall Fzd3 expression pattern was quite comparable across all tumors studied (table 1).

### Heterogenous expression of Fzd5 in non-melanoma skin cancer

**Figure 3:**
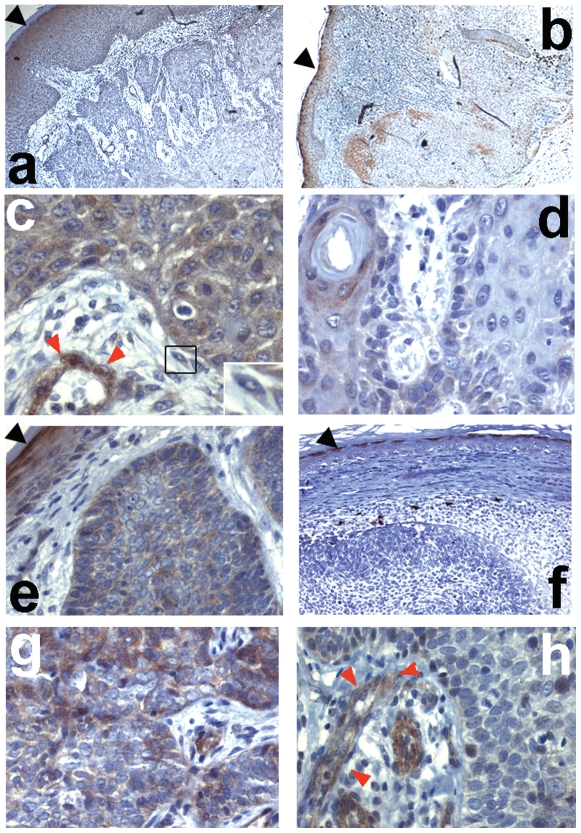
Localization of Fzd5 in non-melanoma skin cancer. Immunohistochemistry of SCC (a–d), or BCC (e–h), in each case showing an example of tumors exhibiting high (SCC: a,c; BCC: e,g) or low (SCC: b,d; BCC: f,h) Fzd5 expression. Staining intensities in the tumors can be directly compared to staining intensities of Fzd5 in the granular layer of the epidermis (black arrowheads). Panels a,b,e,f are shown at 40× and c,d, g,h at 200× magnification. The inset on the lower right of (c) shows a tumor-associated fibroblast. Red arrows denote blood vessels.

Fzd5 is another recognised Wnt5a receptor. Since we previously found that Fzd5 expression in adult epidermis is restricted to the high granular layer ([14] and figure 3a, black arrow), indicative of an expression pattern governed by differentiation, we studied whether Fzd5 expression in cancers reflected tumor differentiation status. In contrast to Fzd3, Fzd5 did not exhibit focal intracellular distribution and was variable between individual tumors. While the majority of SCC tumors exhibited moderate-to-strong Fzd5 expression (fig. 3a,c), 3 of 11 tumors showed weak-to-absent staining (fig. 3b,d). Of note, these variations were not related to tumor differentiation status. Only two BCC samples exhibited strong Fzd5 expression (fig. 3g), while it was low or undetectable in the majority (fig. 3f,h; table 1). As with Fzd3, tumors that did express Fzd5 exhibited Fzd5-positive regions alternating with Fzd5-negative regions (fig. 3a). By contrast, tumor – associated endothelial cells consistently exhibited strong Fzd5 expression (fig. 3c,h). Tumor-associated fibroblasts were weak to moderately positive for Fzd5 (fig. 3c, inset). Thus, while Fzd5 expression is variable in non-melanoma skin cancer cells, its expression level in tumor-vessels is consistent with a role of this receptor in mediating Wnt5a-dependent inflammatory pathways, consistent with previous reports [20], [21].

### Wnt5a and Fzd receptors are expressed by non-overlapping tumor cell subsets

**Figure 4:**
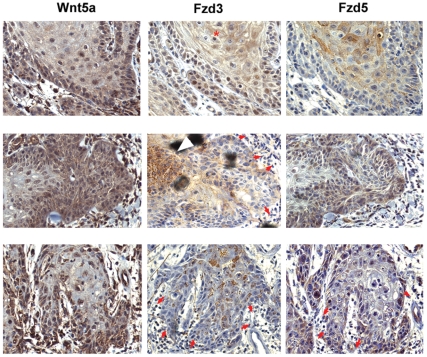
Spatial relationship of Wnt5a, Fzd3, and Fzd5 in squamous cell carcinoma. Serial sections of three paraffin embedded SCC tumor samples (top, middle, bottom row, respectively) were stained for Wnt5a, Fzd5, Fzd3, respectively, as described in Methods, and shown at 200× magnification. Red asterisk denotes artificial nuclear staining possibly due to antigen retrieval conditions. Red arrows denote boundaries of tumors, pointing toward stroma.

**Figure 5:**
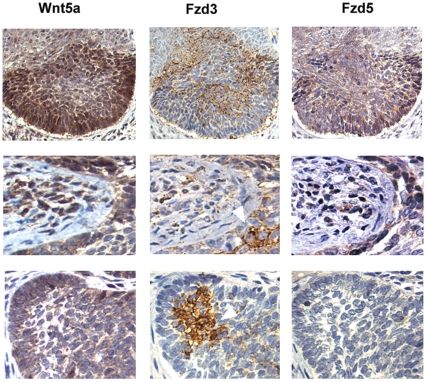
Spatial relationship of Wnt5a, Fzd3, and Fzd5 localization in basal cell carcinoma. Immunohistochemistry of serially cut samples stained for Wnt5a, Fzd5, or Fzd3 as indicated, magnification: 100× (top row), 200× (middle, bottom rows).

We next studied the spatial relationship of Wnt5a, Fzd3, and Fzd5 in individual tumor samples. To this end, we determined staining intensities of these proteins in serial sections of individual tumors, respectively, since antibodies suitable for co-immunofluorescence in paraffin-embedded samples were unavailable. As shown in figure 4, Wnt5a was predominantly expressed on tumor margins in SCC (as well as in tumor associated stroma). By contrast, Fzd3 localized to cells within the tumor mass, sometimes forming nest-like Fzd3-positive tumor-domains (middle panel, white arrow head) and, as described above, exhibiting focally polarised intracellular distribution. Fzd5 exhibited heterogenous intra-tumor distribution, being localised to intra-tumor clusters (top), weak/diffusely at the edge (bottom), or homogenously throughout (middle). Analogous arrangements were observed in BCC, with Wnt5a being most strongly expressed at the leading edge, as well as in tumor-associated stroma (figure 5, left panels), while Fzd3 showed polarised expression within the tumor (figure 5, top middle), or in Fzd3-positive nests (white arrow heads). Fzd5 was weak or absent in most BCCs (bottom right, cf. table 1). Where expressed, Fzd5 exhibited diffuse intra-tumor localisation (upper right), or at the tumor edge (middle right). Taken together, the data show that Wnt5a and its receptors are expressed by non-overlapping sub-populations. The strong expression of Wnt5a at the leading edge, as well as in tumor-surrounding stroma cells, in conjunction with the polarised expression of Fzd3 within the tumor mass suggest that Wnt5a gradients project into the tumor to enhance motility in distinct subpopulations.

### Wnt5a concentration gradients enhance directional motility of keratinocytes

**Figure 6:**
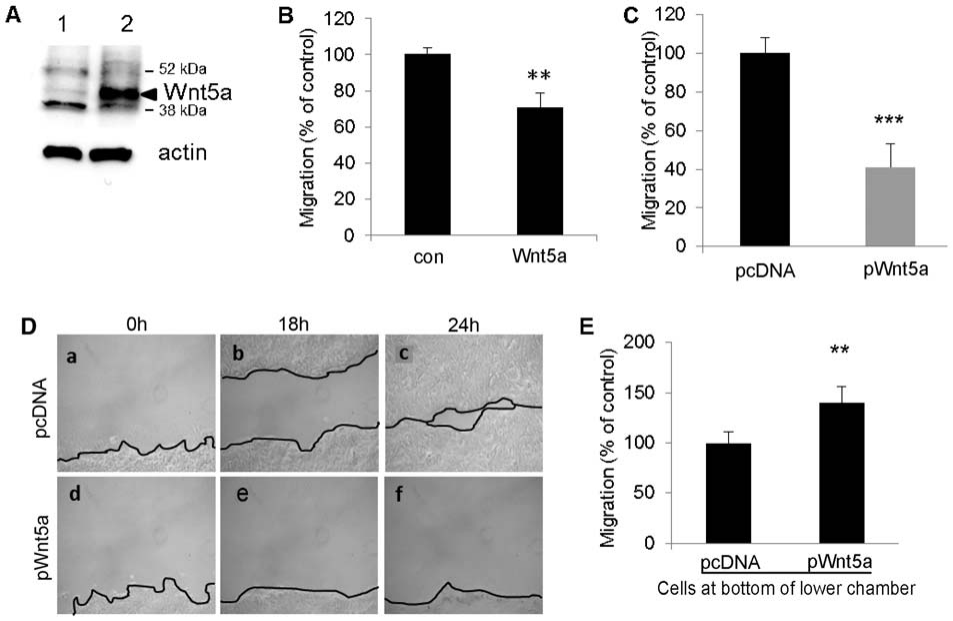
Wnt5a inhibits keratinoctye migration when present in homogenous concentration, but acts as chemoattractant when present as gradient. **A.** Expression of endogenous and recombinant Wnt5a in whole cell lysates of stably transfected Wnt5a-overexpressing HaCat or control (HaCat-pcDNA) cells verified by western blot. **B.** Non-Wnt5a overexpressing HaCat-pcDNA cells were seeded in the upper chamber of a Transwell in 0.1% BSA DMEM in the absence or presence of recombinant Wnt5a at 1 µg/ml, as indicated in the figure. The lower chamber was filled with 600 µl DMEM containing 5% FCS as chemoattractant. Results are expressed as percentage of migrating cells when HaCat-pcDNA were seeded in 0.1% BSA DMEM only. The results shown represent mean ± s.d. of two independent experiment, each performed in triplicate, *p≤0.05. **C.** Comparison of Wnt5a-overexpressing and pcDNA control cell migration. Cells suspended in 0.1% BSA DMEM were seeded in the upper chamber. The lower chamber were filled with 600 µl DMEM containing 5% FCS as chemoattractant. Migration was assessed at 18 h using a colorimetric assay. Results are expressed as percentage of HaCat-pcDNA migrating cells. Results shown represent mean ± s.d. of n = 4 independent experiment, each performed in triplicate, *** p≤0.001. **D.** Scratch wound assay performed on mitomycin-C treated cells. During migration, HaCat-pcDNA (a, b, c), or Wnt5a-overexpressing cells (d, e, f) were maintained in DMEM containing 10% FCS. Pictures were taken just after the scratch was made (0 hrs) (a and d), as well as 18 h (b and e) and 24 h later (c and f). **E.** Migration of HaCat-pcDNA control cells in the presence of a Wnt5a concentration gradient. Wnt5a-overexpressing or pcDNA HaCat cells were seeded in the bottom wells of Transwell plates. Immediately before adding the inserts containing HaCat-pcDNA cells in the upper chamber, the media in the bottom wells was replaced to remove pre-secreted Wnt5a. Migration was assessed at 18 h. Results are expressed as percentage of HaCat-pcDNA migrating cells. Results shown represent mean ± s.d. of n = 3 independent experiments, each performed in triplicate, *** p≤0.001.

The data summarised above suggested Wnt5a concentration gradients may be important for its effect on cell motility. To test this hypothesis functionally, we used human HaCat keratinocytes as a model. We stably transfected HaCat cells with a Wnt5a-expressing vector, or empty control vector (termed HaCat pcDNA). Expression of recombinant Wnt5a was verified by western-blot (fig. 6A). First, we assessed directed cell migration in a two-chamber Transwell assay. As shown in figure 6B, when recombinant Wnt5a was added directly to HaCat-pcDNA keratinocytes in the upper chamber, thus present in a homogenous concentration around migrating cells, it inhibited chemotactic migration toward 5% FCS present in the bottom well. Likewise, chemotactic migration was significantly reduced in cells overexpressing Wnt5a relative to non-overexpressing cells (fig. 6C). An analogous result was obtained in a short term (6 hours) migration assay using either 5% FCS or EGF as chemoattractant (fig. S2A). Moreover, in scratch assays, the migration of Wnt5a-overexpressing HaCat cells in 10% FCS DMEM (fig. 6D) toward the scratch edge was greatly reduced compared to HaCat-pcDNA cells (similar results were found when using 1% FCS as chemoattractant, fig. S2A). Thus, keratinocyte migration is inhibited when Wnt5a surrounds the cells at homogenous concentration. By contrast, when Wnt5a-secreting HaCat cells were seeded at the bottom of a Transwell chamber, thereby establishing an upward Wnt5a concentration gradient, migration of non-Wnt5a overexpressing Hacat cells seeded in the top chamber toward the Wnt5a source was significantly enhanced (fig. 6e). These data show that human keratinocytes are induced to migrate toward a Wnt5a-gradient, but that non-gradient Wnt5a decreases motility toward other chemoattractants.

### Increased Wnt5a expression in cutaneous SCC is accompanied by repression of canonical Wnt signalling and downregulation of signalling inhibitors

**Figure 7:**
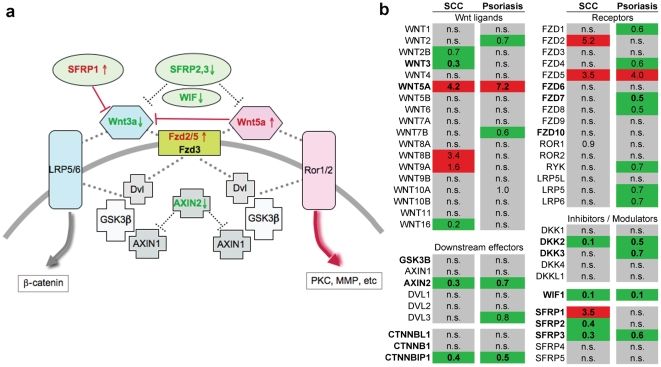
Specific upregulation of non-canonical Wnt signallling and repression of canonical Wnt signalling in SCC. (a) Cartoon illustrating functional relationships between Wnt signalling components listed in tables 2 and 3. Red: upregulated, Green: down-regulated. Large dotted lines represent protein binding. (b) Specific dysregulation of SFRP1 and SFRP2 in invasive SCC, but not psoriasis. Fold-dysregulation of transcripts in psoriasis plaques was calculated as described previously [19] and aligned to the SCC data set described in tables 2 and 3. Color coding and bold type set as in table 2. “n.s.”: not significant.

Since canonical and non-canonical pathways cross-inhibit each other, the effect of Wnt5a signalling *in vitro* is dependent on the relative abundance of other ligands, modulators, receptors, and downstream effectors in the Wnt signalling network. We therefore performed a comprehensive analysis of the expression of Wnt-signalling components in primary invasive cutaneous squamous cell carcinoma. As shown in table 2, Wnt5a was the most significantly upregulated of all wnt ligands (four-fold, p = 8×10^−6^), independenly confirming the immunohistochemistry data. By contrast, the most highly expressed canonical Wnt member, Wnt3a, is significantly down-regulated, thereby alleviating competitive antagonism for Wnt5a at the receptor level. (Another canonical Wnt ligand, Wnt8b, is formally upregulated, but appears to be expressed at much lower total levels, Table 2). Among recognised Wnt5a-binding frizzled receptors, Fzd2 and Fzd5 are upregulated, albeit at marginal statistical significance (Table 3). Among extracellular Wnt antagonists SFRP1 is upregulated, consistent with further repression of canonical Wnt signalling (see below, Discussion). DKK2, specific for canonical Wnt members, is also repressed but the expression of the much higher expressed DKK1 is unaltered. By contrast, antagonists targeting both canonical and non-canonical Wnts, Wif and SFRP2/3, as well as the key intracellular signalling antagonist Axin2, are all significantly downregulated. These changes are graphically summarised in fig. 7a. Collectively, they suggest that the upregulation of Wnt5a in invasive SCC is part of a set of changes acting synergistically to boost non-canonical, but repress canonical Wnt signalling (see below, Discussion).

**Table 2:** Expression of Wnt – ligands in cutaneous SCC.^1^

Gene	expression level^2^		fold change^3^	p value
	con	tumor		
Canonical Wnts^4^				
WNT1	10	22	n.s.	
WNT2	98	266	n.s.	
WNT2	98	266	n.s.	
WNT2B	195	134	n.s.	
**WNT3**	2608	886	0.3	6.E-06
WNT8A	56	85	n.s.	n.s.
WNT8B	17	58	3.4	0.004
WNT10A	301	317	n.s.	
WNT10B	115	113	n.s.	
Non-canonical Wnts				
WNT4	97	97	n.s.	
**WNT5A**	696	2915	4.2	8.E-06
WNT5B	123	202	n.s.	n.s.
WNT6	107	143	n.s.	n.s.
WNT7A	61	95	n.s.	n.s.
WNT7B	274	244	n.s.	n.s.
WNT11	61	132	n.s.	
Unclassified Wnts				
WNT9A	128	199	1.6	0.003
WNT9B	24	27	n.s.	
WNT16	510	127	0.2	2.E-04

1 Data shown (extracted from [18]) represent n = 10 paired samples of moderately differentiated cutaneous invasive SCC from immunocompetent patients. BOLD print: fluorescence intensity (arbitrary units) of >1000 in either control or tumor.

2 Expression levels are the inverse log2-transform of raw fluorescence intensity data provided by [18]. Where multiple probes per gene were present on the array, the probe yielding the highest fluorescence value is shown. For none of the genes listed in the table was there an inverse or significant fold-change for alternative probes.

3 Fold-changes between SCC and control samples with a p-value <0.02 are shown.

4 Classified according to [27].

**Table 3:** Expression of Wnt – signalling components in cutaneous SCC.^1^

Gene	expression level^2^		fold change^3^	p value
	con	tumor		
**Receptors**				
FZD1	152	139	n.s.	
FZD2	94	485	5.2	0.02
FZD3	160	102	n.s.	
FZD4	35	50	n.s.	
FZD5	16	57	3.5	0.021
FZD6	2058	1737	n.s.	
FZD7	708	513	n.s.	
FZD8	443	364	n.s.	
FZD9	53	37	n.s.	
FZD10	771	520	n.s.	
**Co-receptors**				
ROR1	222	193	n.s.	
ROR2	80	59	n.s.	
RYK	177	156	n.s.	
LRP5L	107	88	n.s.	
LRP5	160	161	n.s.	
LRP6	240	174	n.s.	
**Extracellular inhibitors/modulators**				
DKK1	90	552	n.s.	
**DKK2**	1310	95	0.1	5.E-12
DKK3	5640	4304	n.s.	
DKK4	34	54	n.s.	
DKKL1	30	36	n.s.	
**WIF1**	3405	247	0.1	1.E-04
**SFRP1**	773	2707	3.5	0.003
**SFRP2**	12057	4286	0.4	4.E-04
**SFRP3/FRZB**	1362	350	0.3	1.E-04
SFRP4	46	77	n.s.	
SFRP5	39	71	n.s.	
**Downstream signalling components**				
AXIN1	219	258	n.s.	
AXIN2	1280	341	0.27	5.4E-11
**DVL1**	925	1197	n.s.	
DVL2	522	567	n.s.	
DVL3	106	163	n.s.	
**GSK3B**	1270	2055	n.s.	
**CTNNBL1**	1274	1418	n.s.	
**CTNNB1**	4018	2771	n.s.	
**CTNNBIP1**	5285	2245	0.4	2.E-04

1 Data shown are as detailed in Table 2. BOLD print indicates fluorescence intensity (arbitrary units) of >1000 in either control or tumor sample.

2 Expression levels are the inverse log2-transform of the raw fluorescence intensity in [18]. Where multiple probes per gene were present on the array, the probe yielding the highest fluorescence value is shown. For none of the genes listed in the table was there an inverse or significant fold-change for alternative probes.

3 Fold-changes between SCC and control samples with a p-value <0.02 are shown.

### Concurrent inverse transcriptional regulation of Wnt5a and Wnt3a distinguishes SCC from non-invasive benign hyperproliferation

The transcriptional upregulation of Wnt5a itself is unlikely to cause invasiveness, since it is also strongly upregulated in psoriasis, a hyperproliferative but non-invasive disorder [14]. We therefore sought to identify additional factors turning physiological Wnt5a action into an enhancer for invasive migration. To this end we compared gene expression of wnt signalling components in SCC with psoriasis. In both conditions, the respective pathological state is compared to healthy control skin. The relative level of gene expression, expressed as rank level, was found to correlate well between the control skin samples in both data sets, respectively, indicating that dysregulation of genes detected in either condition occurs relative to a similar control (Fig. S3). Figure 7b shows a color-coded dysregulation heat map of the Wnt-signalling components listed in tables 2 and 3 for SCC vs. psoriasis. In confirmation of our previous findings [14], Wnt5a and Fzd5 are also upregulated in psoriasis. Likewise, the downregulation of the canonical wnt inhibitor DKK2, CTNNBIP1 (ICAT), Axin2, as well as FRZB (SFRP3) is common to SCC and psoriasis. However, the repression of Wnt3 as well as the dysregulation of SFRP1 and SFRP2 are only found in invasive cutaneous SCC, but not psoriasis.

### Lack of nuclear β-catenin staining and weak Axin2 protein expression confirm down-regulated canonical Wnt-signalling in SCC and BCC

**Figure 8:**
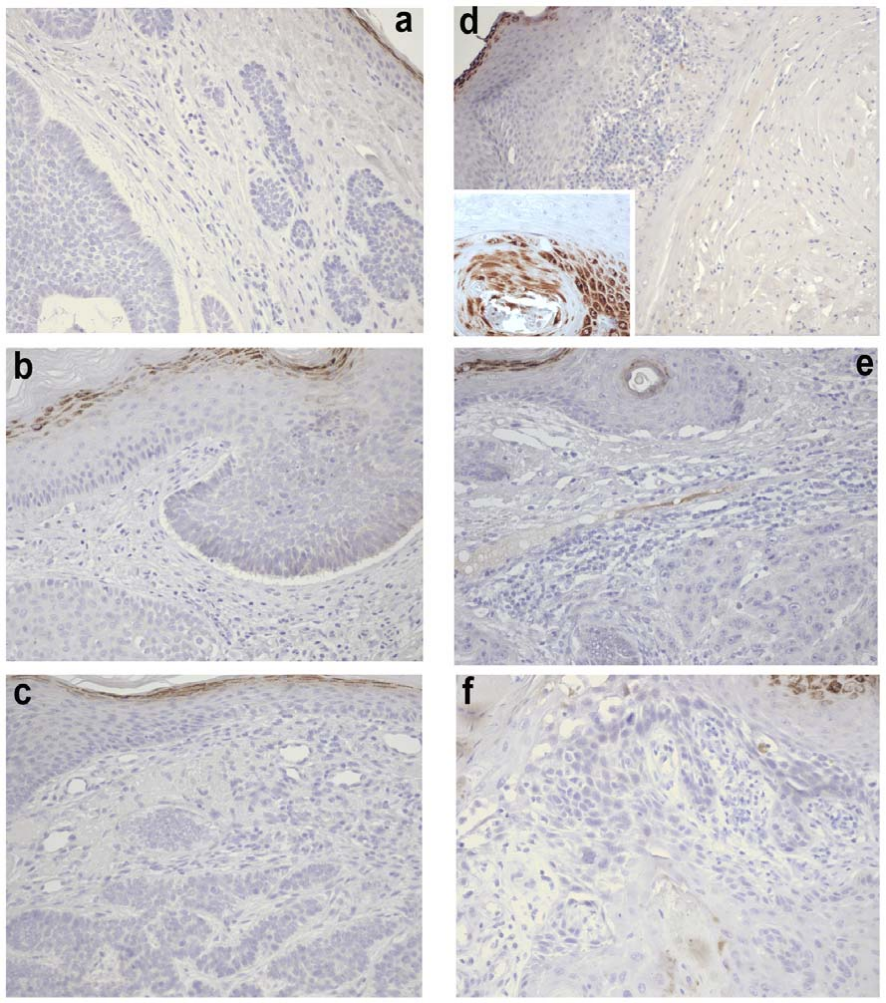
Lack of nuclear β-catenin in SCC and BCC. Immunohistochemistry of three BCC (a–c) and SCC (d–f) samples stained with an antibody specific for activated β-catenin. Note strong nuclear β-catenin confined to the granular layer of the epidermis in each sample, as well as in a magnified hair follicle immediately below SCC cells (inset in d). All samples shown at 100× magnification, inset at 400×.

**Figure 9:**
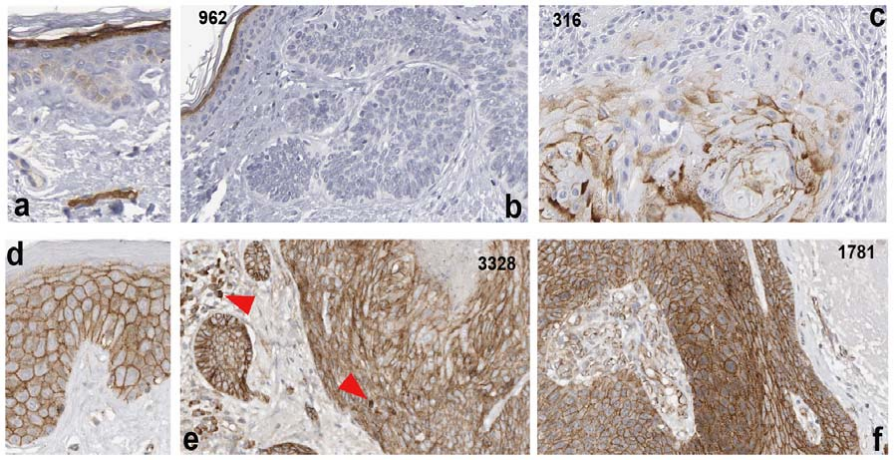
Immunohistochemical detection of β-catenin in BCC (b, e), and moderately differentiated SCC (c, f) samples at the ProteinAtlas repository (see main text). Samples were stained either with an antibody specific for activated non-phosphorylated β-catenin (top) or pan-β-catenin (bottom). Images in (a) and (d) show the β-catenin distribution observed with the respective antibody. Note that strong nuclear β-catenin is confined to the granular layer of the epidermis.

In order to obtain further independent evidence for the activation status of canonical Wnt-signalling we performed immunohistochemistry of β-actin, using an antibody specific for activated (Ser38/Thr41 dephosphorylated) β - actin. As shown in figure 8, nuclear β-catenin was abundant in the granular layer of the epidermis but absent from either SCC or BCC tumors. This was true for all SCC (n = 12) and BCC (n = 7) samples studied. We furthermore took advantage of the publicly available tissue array repository available at the ProteinAtlas website. This database contains immunohistochemistry data varying in quality depending on the reagents used. In the case of β-catenin, the website contains series stained with four different antibodies, three of which have been extensively validated (see Data S1). IHC images generated using either the same antibody specific for activated β-catenin or antibodies detecting total β-catenin revealed the presence of membrane-located β-catenin but the consistent absence of nuclear β-catenin in 12/12 SCC and in 11/BCC using four different antibodies (fig. 9, Data S1). Finally, we also mined the data at ProteinAtlas available for Axin2. Even though the level of validation of this antibody is not quite as robust as for the β-catenin antibodies, the available data strongly suggest that Axin2 fails to accumulate in either SCC or BCC (figure S6). Thus, protein level data are consistent with the expression profiling data, suggesting repression of canonical Wnt signalling.

## Discussion

Numerous studies have suggested a role of Wnt5a in cancer invasion (e.g. [16], [22]–[25]. Nonetheless, the role of Wnt5a in cancer is controversial, some reports suggesting that Wnt5a may act as tumor suppressor by antagonising canonical Wnt signalling (reviewed in [26]). On the other hand, both canonical and non-canonical Wnt signalling, though mutually antagonistic, appear to act complementary in different stages of colorectal cancer development, Wnt5a being upregulated in the later invasive stage [27]. One crucial aspect of Wnt5a action on cancer cell motility is the presence of concentration gradients. When added directly to cell medium, Wnt5a inhibits cell migration. This observation (fig. 6) has been made by others previously and taken as evidence that Wnt5a blocks invasion (e.g. [28]). However, when applied as gradient (fig. 6e), Wnt5a clearly stimulates directed motility [15]. The present data (strong expression of Wnt5a at the edge and in surrounding stroma, focal polarised intracellular distribution of Fzd3 within the tumors) suggests the existence of Wnt5a gradients acting from the tumor margin (as well as Wnt5a expressing stroma cells, fig. 1b,d) on cells within the tumor, thereby increasing chemotactic motility into adjacent tissue.

In terms of receptor binding, Wnt5a can bind a number of Fzd receptors. The present data suggest that Fzd5 is unlikely to mediate invasion related effects. Thus, in contrast to Fzd3, Fzd5 is weak or absent in 3/12 SCC and 7/9 BCC. In addition, we previously found Fzd5 also upregulated in psoriasis. However, Fzd5 may mediate inflammatory responses triggered by Wnt5a secreted by tumor-associated stroma or endothelial cells such as inflammatory cytokine production [20], [29]. A further recognised Wnt5a receptor, is Fzd2. Although we could not study Fzd2 by immunohistochemstry for want of a suitable antibody, we did find Fzd2 upregulated by expression profiling in SCC, but not in psoriasis (table 3, fig. 7b). Fzd2 has been shown to enhance invasiveness in an autocrine manner by controlling focal adhesion dynamics at the leading edge [30], a mechanism in line with the distribution of Wnt5a at the leading edge found here in SCC and BCC. Functional studies will be required to determine if Fzd2 mediates Wnt5a-driven invasiveness in these cancer types.

The comparison of wnt-signalling related expression in SCC versus the non-invasive hyperproliferative state in psoriasis (fig. 7b) confirms that the inverse regulation of non-canonical and canonical Wnt signalling is specific for the invasive phenotype. Similarly, the non-invasive, pro-inflammatory response in lung epithelia to acutely increased mechanical pressure is characterised by concurrent activation of *both* types of Wnt signalling [31]. Our findings are in confirmation of a previous study which failed to detect nuclear β-catenin accumulation in SCC [32] whereas this is increased in psoriasis [33]. One previous study did report downregulation of canonical Wnt signalling in psoriasis [34]. However, based on Axin2 as prototypical target of this pathway, the changes observed are minor (fig. 3b therein). Interestingly, the specific activation of β-catenin in the granular layer, as well as Axin2, shown here may explain the slight downregulation of canonical Wnt signalling noted in psoriasis, since this layer fails to be formed in that disease. While downregulation of SFRP3 also occurs in psoriasis, SFRP3 is unable to bind to Wnt1 directly [35], thus placing it outwith the canonical Wnt signalling pathway.

The data presented here suggest that SFRP proteins, in particular SFRP2, exhibit very high constitutive expression in normal skin (table 3), in confirmation of a recent report showing strong SFRP2 protein staining in skin observed by immunohistochemistry [36]. The physiological role of SFRP2 in the skin include both modulation of Wnt signalling as well as Wnt-independent functions. First, the spatial arrangement of Wnt5a versus SFRP2 suggests that SFRP2 shapes Wnt gradients by acting as a diffusion barrier analogous to its role in development [7], [37]. Thus, in the epidermis, Wnt5a is most strongly expressed in the basal layer, while SFRP2 is highly expressed in suprabasal cells [36]. In the hair follicle, Wnt5a is massively expressed in the dermal papilla [14] while it forms a ring-like enclosure in the inner root sheath [38]. In both structures, excess SFRP2 is thus poised to maintain a unidirectional Wnt5a concentration gradient. Second, independent of Wnt, SFRP2 activates pro-collagen proteases such as BMP-1, thereby enhancing collagen maturation [39]. Thus, the reduction of SFRP2 in SCC decreases collagen fibril deposition in the tumor stroma as it does in other tissue [39], facilitating invasive cell migration. Both wnt-dependent and wnt-independent functions of SFRP2 therefore counter tissue invasion. That the massive downregulation of SFRP2 in SCC is clinically relevant, is additionally strongly suggested by numerous reports of epigenetic SFRP2 silencing in invasive cancers (see below).

Concomitant to the repression of SFRP2, invasive SCC is marked by strong upregulation of SFRP1 (table 3). Several lines of evidence suggest that both of these changes in fact synergise to promote hyperactive Wnt5a signalling. First, SFRP1 has been shown to bind canonical Wnt1 but is unable to bind Wnt5a [40] and also antagonises Wnt1 function but not Wnt5a function in Xenopus development [41], suggesting that SFRP1 upregulation further represses canonical Wnt signalling. Second, while SFRP2 increases sensitivity toward apoptosis, SFRP1 has the opposite effect [42]. Third, SFRP1, but not SFRP2, is a potent angiogenic factor independent of Wnt signalling, suggesting its upregulation enhances tumor vascularisation [43], [44]. Fourth, and most importantly, only SFRP2, but not SFRP1 silencing by promoter methylation was observed in oral SSC [45] and gastric cancer [46], and promoter methylation is significantly higher in SFRP2 than SFRP1 in cervical cancer [47], as well as in cervical adenocarcinoma [46], [48].

In conclusion, we here show that Wnt5a is overexpressed in non-melanoma skin cancer, localises to the invasive tumor edge, and directs gradient – dependent motility of keratinocytes in vitro. Our data suggest that concurrent upregulation of Wnt5a and repression of Wnt3a as well as SFRP2 is sufficient to drive tissue invasion *in vitro*, a hypothesis which is testable using our previously established SCC-based *in vitro* carcinogenesis model [49]. Finally, our results establish cutaneous non-melanoma skin cancer as model to analyse dysequilibrium between canonical and non-canonical Wnt signalling.

## Supporting Information

Data S1 **Expression of β-catenin in SCC and BCC.** Section 1: Absence of activated β-catenin in 12/12 SCC and 11/11 BCC tumors in the ProteinAtlas database. Section 2: Literature- review of published IHC staining data on β-catenin expression in SCC and BCC, suggesting consistent reduction of β-catenin in SCC. Section 3: critical appraisal of the data on β-catenin in SCC published in Malanchi et al, Nature 2008. Section 4: critical appraisal of the data on β-catenin in BCC published in Yang et al, Nature Genetics, 2009.
(PDF)

Figure S1 **Immunohistochemistry of Fzd3 in normal adult skin and anagen hair follicle, performed as detailed in Methods.** ORS, outer root sheat, Ctx, cortex, DP, dermal papilla. a,b shown at 200× magnification, inserts in c,d at 400×. Arrows denote the polarity line in individual cells pointing away from the Fzd3-pole.
(TIF)

Figure S2 **Non-gradient Wnt5a inhibits chemotactic migration.** A. Short term (6 h) migration assay. Control or Wnt5a-overexpressing HaCat keratinocytes were seeded in the top chamber of a Transwell plate and migration stimulated as detailed in Methods either by DMEM containin 5% FCS, or epidermal growth factor (EGF), as indicated. B. Scratch wound performed on monolayers of mitomycin-C treated cells. HaCat-pcDNA (a, b, c) and wnt5a-overexpressing cells (d, e, f) were maintained in DMEM supplemented with 1% FCS. Pictures were taken just after the scratch was made (0 hrs) (a and d) as well as 18 h (b and e) and 24 h later (c and f).
(TIF)

Figure S3 **Relative levels of gene expression in normal skin are comparable in data sets quantifying gene dysregulation in squamous cell carcinoma (SCC) and psoriasis, respectively.** The fluorescence data from each of the datasets described in Methods and the legend for figure 7 were used to rank the relative fluorescence intensities among the probes yielding the most intensive signal for each gene, respectively. Data shown represent the genes listed in table 2. R^2^ = 0.92. The data show that the control gene expression used to define altered gene expression in either condition is comparable.
(TIF)

Figure S4 **Wnt5a – expression in human epidermis.** Immunohistochemistry using an alternative antibody (mouse monoclonal, clone 3D10) compared to the previously one (mouse monoclonal, order nr. AF645, R&D) confirms the overall expression pattern of Wnt5a, as previously reported: strong expression in the basal layer, strong expression in dermal fibroblasts and subepidermal capillaries. In addition, the samples shown above illustrate some biological variation detected: (i) variable intensity of Wnt5a staining between samples from different individuals (left vs. middle), (ii) additional suprabasal expression in some, but not all keratinocytes in the spinous layer (right), and (iii) discontinuous expression in the basal layer (middle panel). Immunohistochemistry was performed as detailed in Methods, panels shown are at 100× magnification.
(TIF)

Figure S5 **Expression of Wnt5a in SCC and BCC, as detected using an alternative antibody.** Three SCC (top) and BCC (bottom) samples are shown, respectively. In each case, tumor – adjacent epidermis has been inserted to allow assessment of relative staining intensity. Wnt5a staining is strong and varies between homogenous (top right, bottom) and being stronger at tumor edge (top left and middle). Interstingly, the BCC sample on the bottom left suggest existence of tumor subclones with varying Wnt5a expression levels. Magnification 100×.
(TIF)

Figure S6 **Immunohistochemistry of Axin2 in SCC and BCC.** Samples were taken from the repository available at ProteinAtlas.org. Numbers in the tumor samples refer to patient IDs on the website. (a,b): normal anal/vulval skin samples at the website (normal epidermis was not available as separate samples. However, tumor-associated epidermis is shown in panels h,i) show strong expression in the granular layer, paralleling the location of activated β-catenin (see figs. 8,9). Note that despite intensive overall staining, the intracellular localisation of Axin2 appears to be perinuclear/cytoplasmic (insets in panels a,b). Panel C shows specific cytoplasmic staining in goblet cells of colonic mucosa as positive staining control. Further evidence for the validity of the staining results are the much stronger staining observed in colon carcinoma samples vs. normal colonic mucosa in numerous samples at the website, as well as stronger staining seen in ovarian cancer vs. normal ovarian tissue (in confirmation of the data published by Leung et al, “Activation of AXIN2 Expression by β-Catenin-T Cell Factor”, J Biol Chem 2002). Blue arrows: tumor tissue, red arrows: tumor-associated cells affording estimate of relative staining intensity in cluding eccrine glands (d), inflammatory stroma-infiltrate (e,f,g), as well as epidermis (h,i).
(TIF)
