# Supplementary material for: Wnt5a Is Strongly Expressed at the Leading Edge in Non-Melanoma Skin Cancer, Forming Active Gradients, while Canonical Wnt Signalling Is Repressed
Source: PLoS One. 2012 Feb 22;7(2):e31827. doi: 10.1371/journal.pone.0031827 (PMC3285195; doi:10.1371/journal.pone.0031827)
Supplement: Data S1 — Expression of β-catenin in SCC and BCC. Section 1: Absence of activated β-catenin in 12/12 SCC and 11/11 BCC tumors in the ProteinAtlas database. Section 2: Literature- review of published IHC staining data on β-catenin expression in SCC and BCC, suggesting consistent reduction of β-catenin in SCC. Section 3: critical appraisal of the data on β-catenin in SCC published in Malanchi et al, Nature 2008. Section 4: critical appraisal of the data on β-catenin in BCC published in Yang et al, Nature Genetics, 2009. (PDF) [file pone.0031827.s001.pdf]

β-catenin expression in SCC and BCC by immunohistochemistry.

1. Data mining of the ProteinAtlas online resource
2. Other published IHC data of beta-catenin in human cutaneous SCC/ BCC
3. The data in Malanchi et al, Nature 2008
4. The data in Yang et al, Nature Genetics 2009

1. Data from the ProteinAtlas online repository

(http://www.proteinatlas.org/ENSG00000168036/antibody)

Immunohistochemistry data on this site are available obtained using four different antibodies. The data available regarding reagent validity are as follows:

Antibody	Antibody-source	Validation level
Millipore AB19022	Rabbit-polyclonal raised against full-length beta-catenin-GST fusion, non-purified	Qualitatively similar tumor staining to SC 05-665 (below)
Upstate/ Millipore (05-665)	monoclonal raised against N-terminus (amino acids 36-44), specific for non-phosphorylated, active β-catenin	Numerous publications, specific for nuclei in granular layer, colon tumor pattern as expected
Sigma HPA029159	Affinity-purified rabbit polyclonal	Western blot and protein array
Sigma HPA029160	Affinity-purified rabbit polyclonal	Western blot and protein array

Consistent with the different properties of the antibodies, the β-catenin distribution detected using 05-665 is restricted to the granular layer and to the nuclei (images taken from the "normal skin" samples available at the website):

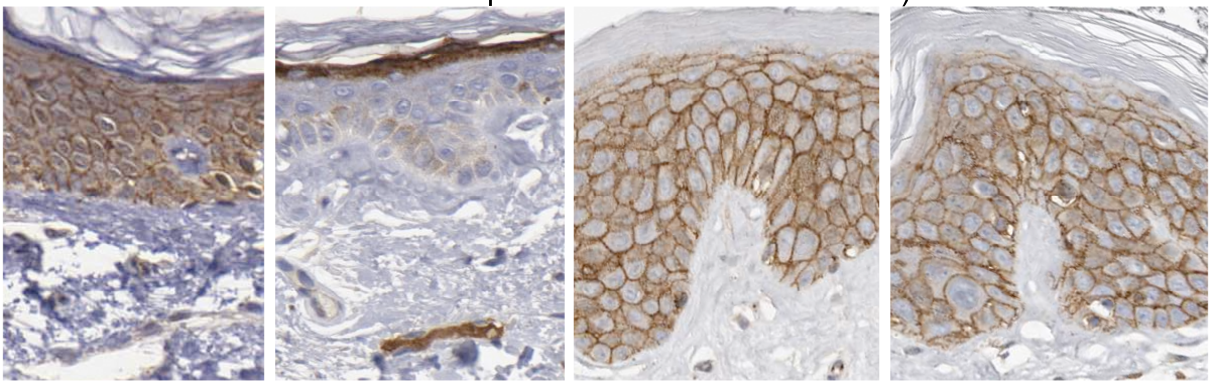

AB19022 05-665 HPA029159 HPA029160

Note that clone 05-665, specific for non-phosphorylated activated β-catenin stains the nuclei in the granular layer as well as some nuclei in the dermis but is only weakly positive for membranous β-catenin.

Ab property	Rabbit polyclonal against full length	specific for activated β-catenin	Affinity purified, N-terminus		Affinity-purified, armadillo-repeat
Ab I.D.	AB19022	05-665	HPA029159		HPA029160
Staining pattern: M = membranous, N = nuclear					
Sample			Sample		
**SCC**					
SCC 654	M weak<br>N absent	M absent<br>N absent	SCC 1781	M strong<br>N absent	M strong<br>N absent
SCC 155	M weak<br>N absent	M absent<br>N absent	SCC 2171	M weak<br>N absent	M weak<br>N absent
SCC 316	M moderate<br>N absent	M moderate<br>N absent	SCC 3261	M weak<br>N absent	M weak<br>N absent
SCC 318	M weak<br>N absent	M absent<br>N absent	SCC 3846	M weak<br>N absent	M weak<br>N absent
SCC 561	M strong<br>N absent	M absent<br>N absent	SCC 1692	M strong<br>N absent	M strong<br>N absent
			SCC 2765	M weak<br>N absent	M weak<br>N absent
			SCC 2111	M moderate<br>N absent	M moderate<br>N absent
**BCC**					
BCC 962	M strong<br>N rare	M absent<br>N absent	BCC 3394	M moderate<br>N rare	M moderate<br>N rare
BCC 674	Not available	M absent<br>N absent	BCC 3328	M moderate<br>N absent	M moderate<br>N absent
BCC 1246	M strong<br>N occasional	M absent<br>N absent	BCC 3407	M strong<br>N rare	M strong<br>N rare
BCC 1279	M strong<br>N rare	M absent<br>N absent	BCC 1983	M moderate<br>N rare	M moderate<br>N rare
BCC 1255	M strong<br>N occasional	M weak<br>N absent	BCC 1335	M strong<br>N rare	M strong<br>N rare
BCC 518	No available	M absent<br>N absent			

In conclusion, 12/12 SCC (each classified as moderately differentiated) and 11/11 BCC in this dataset fail to show any appreciable nuclear β-catenin accumulation, as probed using four different antibodies.

3. Other published IHC data of beta-catenin in human cutaneous SCC/ BCC

A survery of published CTNNB IHC data is provided in the table below. Available data consistently suggest that nuclear β-catenin distribution is reduced in cutaneous SCC, consistent with the present manuscript. With respect to BCC, the data are more variable. Nuclear β-catenin is found in many BCC by some, (strongest data in Yamazaki et al, and El-Bahrawy et al), but not by other investigators (Saldanha et al). It is possible that the cause underlying this discrepancy may be the type of BCC (thus found by Oh et al).

Reference	CTNNB status
Lyakhovitsky A, Am J Dermatopathol. 2004<br>Expression of e-cadherin and beta-catenin in cutaneous squamous cell carcinoma and its precursors.	Reduced in SCC
Brasanac D, Br J Dermatol. 2005.<br>Cyclin A and beta-catenin expression in actinic keratosis, Bowen's disease and invasive squamous cell carcinoma of the skin.	Reduced in SCC versus early AK lesions
Papadavid E, Pignatelli M, Zakynthinos S, Krausz T, Chu AC., J Pathol. 2002 Feb;196(2):154-62.<br>Abnormal immunoreactivity of the E-cadherin/catenin (alpha-, beta-, and gamma-) complex in premalignant and malignant non-melanocytic skin tumours.	Cytoplasmic/nuclear detection of β-catenin (no quantitative data analysis)
Yamazaki F, Br J Dermatol. 2001<br>Immunohistochemical detection for nuclear beta-catenin in sporadic basal cell carcinoma..	14 / 20 BCC show nuclear β-catenin staining
Oh ST, Br J Dermatol. 2011<br>Increased immunoreactivity of membrane type-1 matrix metalloproteinase (MT1-MMP) and β-catenin in high-risk basal cell carcinoma.	Increased nuclear β-catenin in high risk vs nodular BCC
Adegboyega PA, Hum Pathol. 2010<br>Stromal expression of actin is a marker of aggressiveness in basal cell carcinoma.	No data shown
Krahl D, Br J Dermatol. 2010<br>Basal cell carcinoma and pilomatrixoma mirror human follicular embryogenesis as reflected by their differential expression patterns of SOX9 and β-catenin.	β-catenin negative in BCC as opposed to pilomatrixoma
Fukumaru K, J Dermatol. 2007<br>Immunohistochemical comparison of beta-catenin expression by human normal epidermis and epidermal tumors.	β-catenin reduced in BCC and SCC
Saldanha G., Br J Dermatol. 2004<br>Nuclear beta-catenin in basal cell carcinoma correlates	Moderate or strong nuclear β-catenin in

with increased proliferation.	only 7 of 86 BCCs
El-Bahrawy, Br J Dermatol 2003<br>Expression of beta-catenin in basal cell carcinoma.	80 BCCs, nuclear β-catenin in 50 – 70% of samples, depending on subtype.

4. The data presented by Malanchi et al, Nature 2008

One paper, widely noted due to its publication in Nature, explicitly states nuclear accumulation of beta-catenin in human cutaneous SCC. However, this statement is difficult to interpret for the following reasons: First, the only data present to support this conclusion is a single tumor sample (suppl fig.10, for ease shown below).

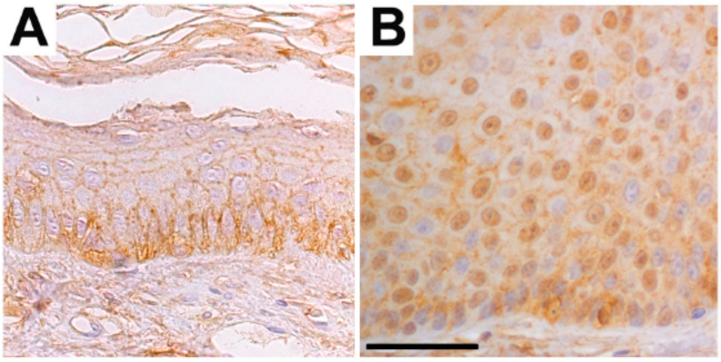

Strikingly, the epidermal distribution of beta-catenin in what is supposed to be normal skin (A) is completely inconsistent with the normal pattern found using any other beta-catenin antibody (see above), in this case showing a selective basal membranous distribution. In addition beta-catenin distribution in the single tumor sample shown was done using an “optimized protocol for detection of nuclear β-catenin.” (legend to suppl.fig.10) which is nowhere to be found. Curiously, although prominent nuclear staining is present in the tumor, almost no membrane staining is observed, which is again at variance with other published data based on many more tumors (see above). Moreover, tumor stroma is not shown.

Finally, the antibody used is not specified. The Methods section details use of “β-catenin (BD Transduction Laboratories, Millipore and ref. 16)”. Reference 16 (Huelsken et al, Cell 2001), in turn, specifies “β-catenin (polyclonal serum, Huelsken et al., 1994, and 7D11, Nanotools” as having been used for immunohistochemistry.
Thus, although published in a well-reviewed journal, the significance of this particular dataset remains uncertain.

5. The data presented in Yang et al, Nature Genetics, 2009

This paper was cited by one of the reviewers of the present manuscript as evidence for beta-catenin activation being critical for BCC progression. Focussing on the data on beta-catenin expression in primary human BCC tissue, a single tumor sample is shown in figure 3, for ease reproduced below:

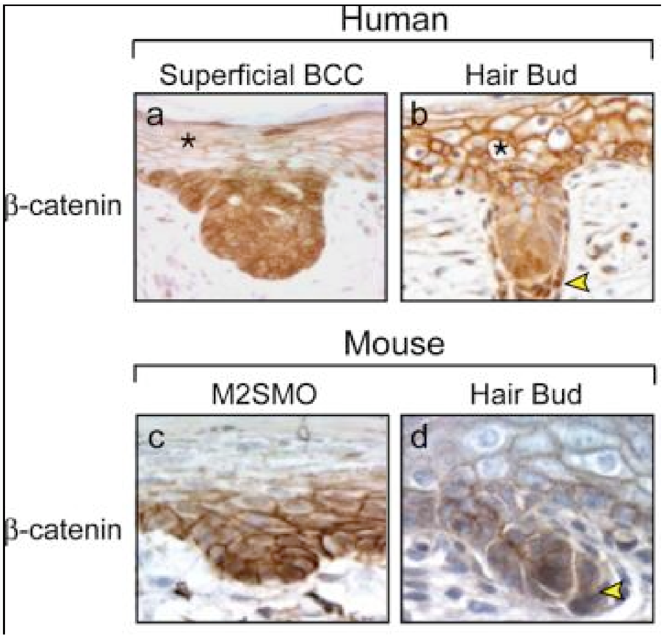

The legend to the figure in the paper interprets the data thus: "nuclear-cytoplasmic β-catenin localization in human superficial BCC [...] and in mouse M2SMO bud cells". However, it is difficult to judge from the figure whether the β-catenin in the single tumor sample is really nuclear or rather cytoplasmic/membranous. Moreover, the membranous epidermal β-catenin staining typically found using pan-β-catenin antibodies (see below) is visible in figure (b) but curiously absent in figure (a), thereby accentuating the tumor "specific" staining. The murine tumor sample certainly looks rather similar to the membrane β-catenin stain visualised in the numerous BCC samples in the ProteinAtlas.

Strikingly, the authors make use of an antibody selective for "non-phoshorylated activated β-catenin". They do not specify the precise product, but only state the source (Upstate). Judging by the product website (Upstate now being part of Millipore) this antibody is identical to the one used in the ProteinAtlas (Anti-Active-β-Catenin (anti-ABC), clone 8E7) which clearly shows the *absence* of nuclear β-catenin in primary BCC samples (see above). In fact, one wonders why the authors apply this antibody to western blotting analysis of the murine tumor sample (figure 3e) but not human BCC samples, either by IHC (for which this antibody is validated)

or western blot. Thus, although the mouse model reported in the paper is very interesting the specific data supplied to describe β-catenin distribution in primary BCC does not appear to be very robust.
