# Supplementary figures and images for: Wnt5a Is Strongly Expressed at the Leading Edge in Non-Melanoma Skin Cancer, Forming Active Gradients, while Canonical Wnt Signalling Is Repressed
Source: PLoS One. 2012 Feb 22;7(2):e31827. doi: 10.1371/journal.pone.0031827 (PMC3285195; doi:10.1371/journal.pone.0031827)

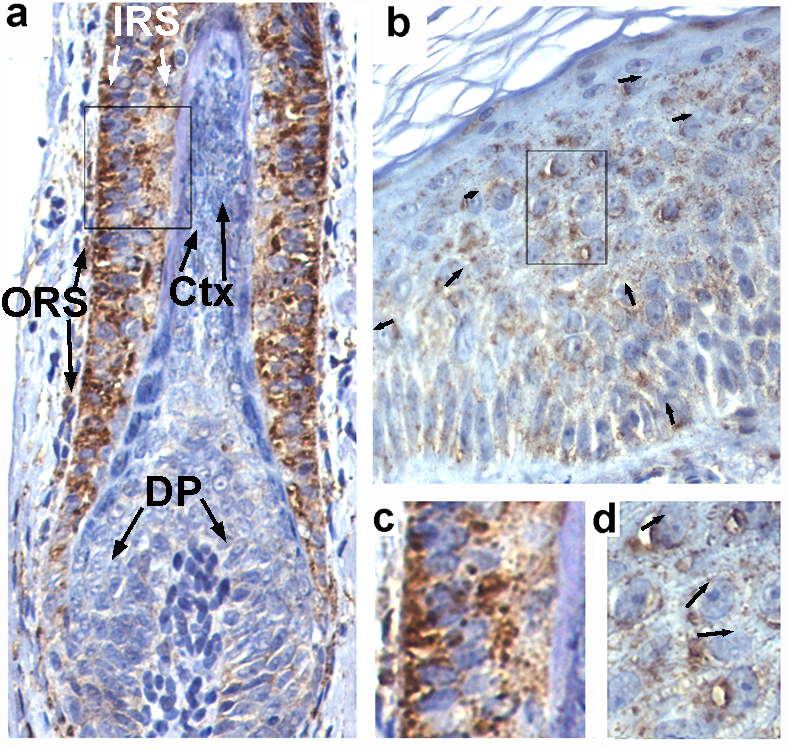

Supplement: Figure S1 — Immunohistochemistry of Fzd3 in normal adult skin and anagen hair follicle, performed as detailed in Methods. ORS, outer root sheat, Ctx, cortex, DP, dermal papilla. a,b shown at 200× magnification, inserts in c,d at 400×. Arrows denote the polarity line in individual cells pointing away from the Fzd3-pole. (TIF) [file pone.0031827.s002.tif]

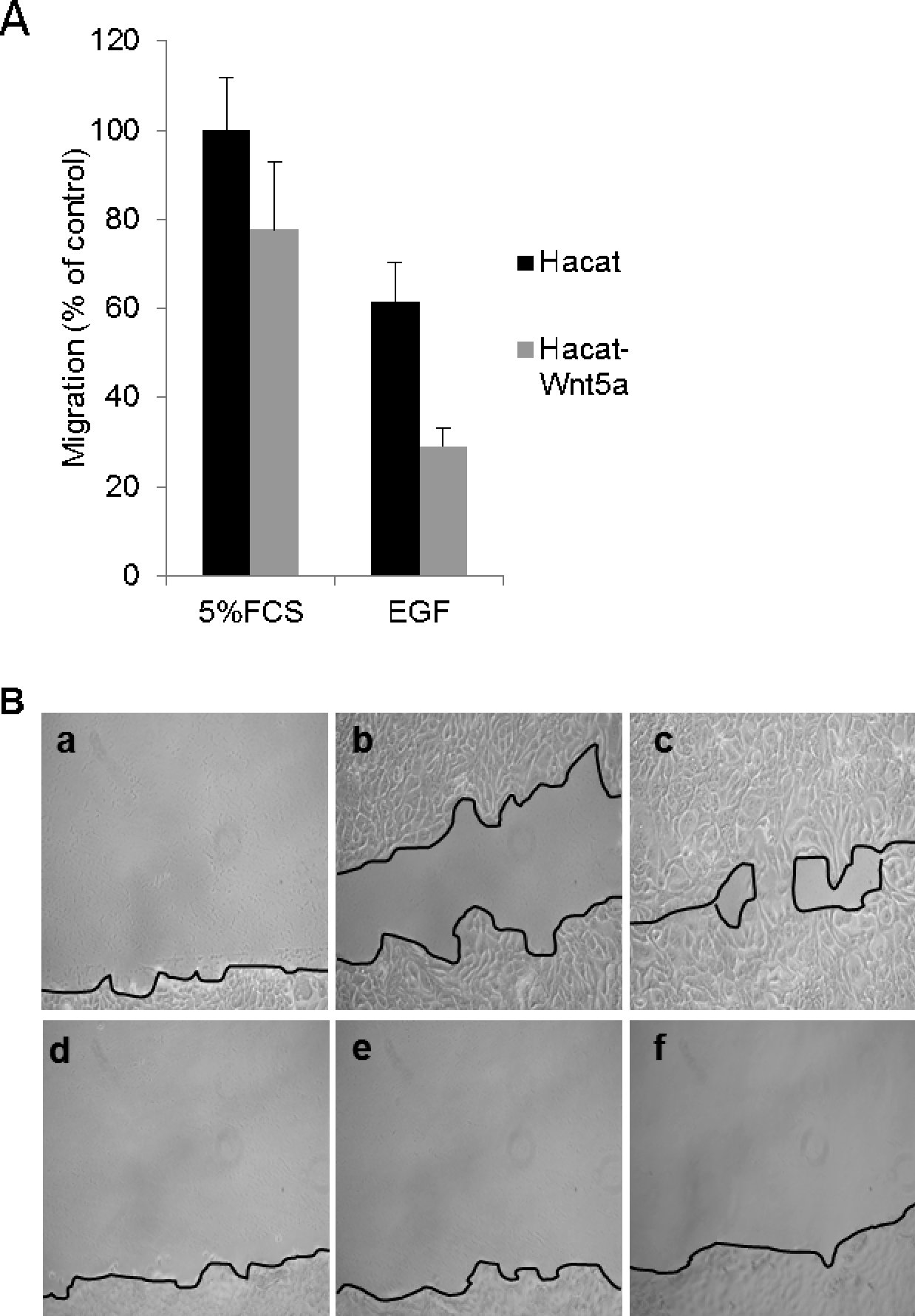

Supplement: Figure S2 — Non-gradient Wnt5a inhibits chemotactic migration. A. Short term (6 h) migration assay. Control or Wnt5a-overexpressing HaCat keratinocytes were seeded in the top chamber of a Transwell plate and migration stimulated as detailed in Methods either by DMEM containin 5% FCS, or epidermal growth factor (EGF), as indicated. B. Scratch wound performed on monolayers of mitomycin-C treated cells. HaCat-pcDNA (a, b, c) and wnt5a-overexpressing cells (d, e, f) were maintained in DMEM supplemented with 1% FCS. Pictures were taken just after the scratch was made (0 hrs) (a and d) as well as 18 h (b and e) and 24 h later (c and f). (TIF) [file pone.0031827.s003.tif]

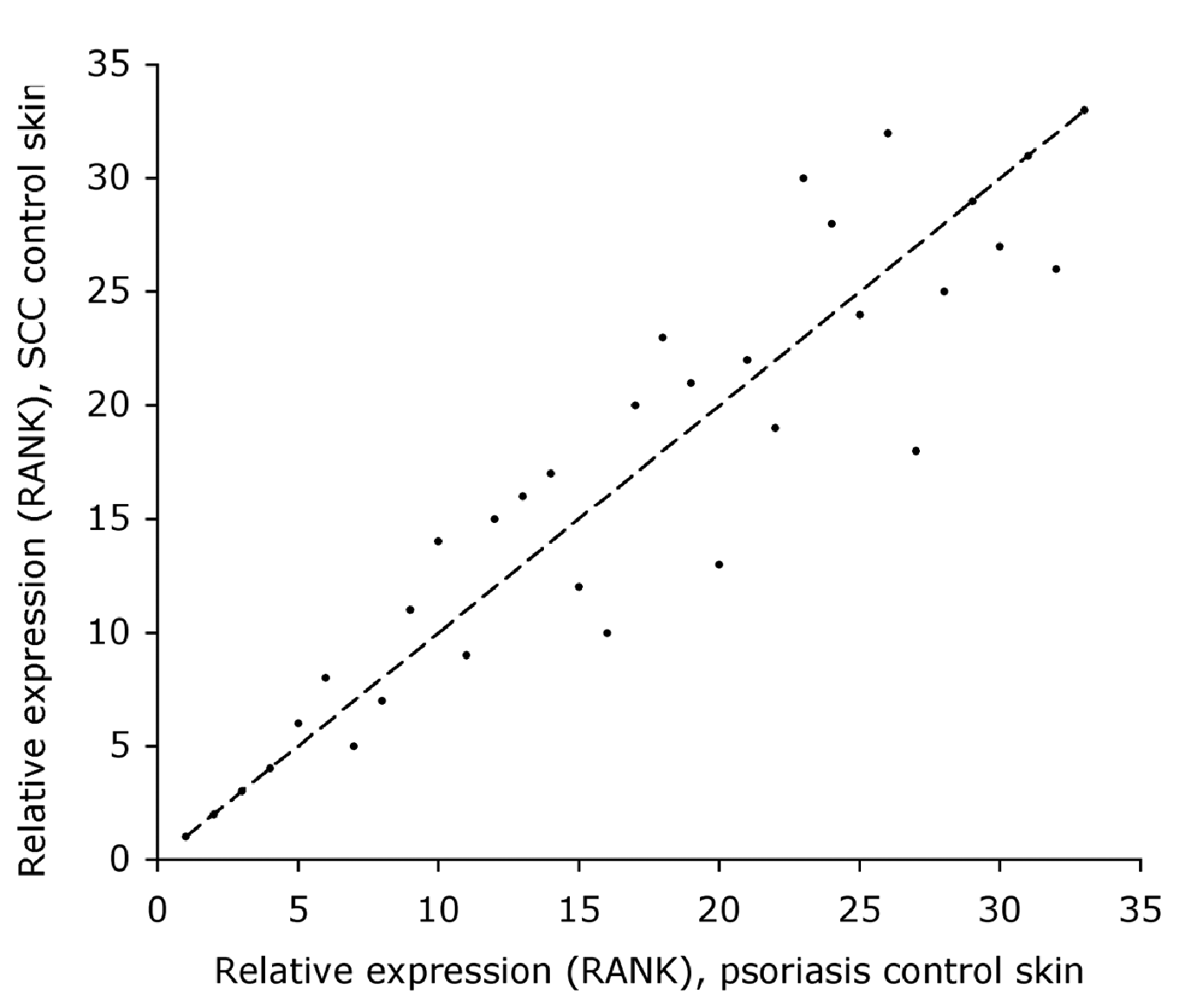

Supplement: Figure S3 — Relative levels of gene expression in normal skin are comparable in data sets quantifying gene dysregulation in squamous cell carcinoma (SCC) and psoriasis, respectively. The fluorescence data from each of the datasets described in Methods and the legend for figure 7 were used to rank the relative fluorescence intensities among the probes yielding the most intensive signal for each gene, respectively. Data shown represent the genes listed in table 2. R2 = 0.92. The data show that the control gene expression used to define altered gene expression in either condition is comparable. (TIF) [file pone.0031827.s004.tif]

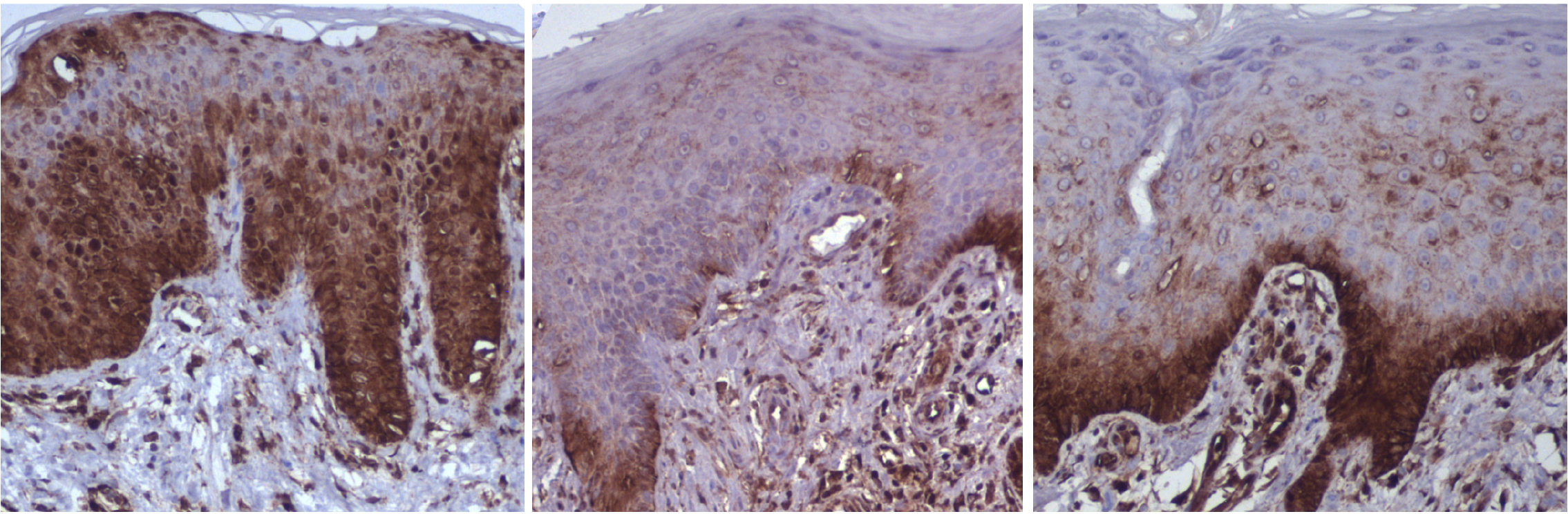

Supplement: Figure S4 — Wnt5a – expression in human epidermis. Immunohistochemistry using an alternative antibody (mouse monoclonal, clone 3D10) compared to the previously one (mouse monoclonal, order nr. AF645, R&D) confirms the overall expression pattern of Wnt5a, as previously reported: strong expression in the basal layer, strong expression in dermal fibroblasts and subepidermal capillaries. In addition, the samples shown above illustrate some biological variation detected: (i) variable intensity of Wnt5a staining between samples from different individuals (left vs. middle), (ii) additional suprabasal expression in some, but not all keratinocytes in the spinous layer (right), and (iii) discontinuous expression in the basal layer (middle panel). Immunohistochemistry was performed as detailed in Methods, panels shown are at 100× magnification. (TIF) [file pone.0031827.s005.tif]

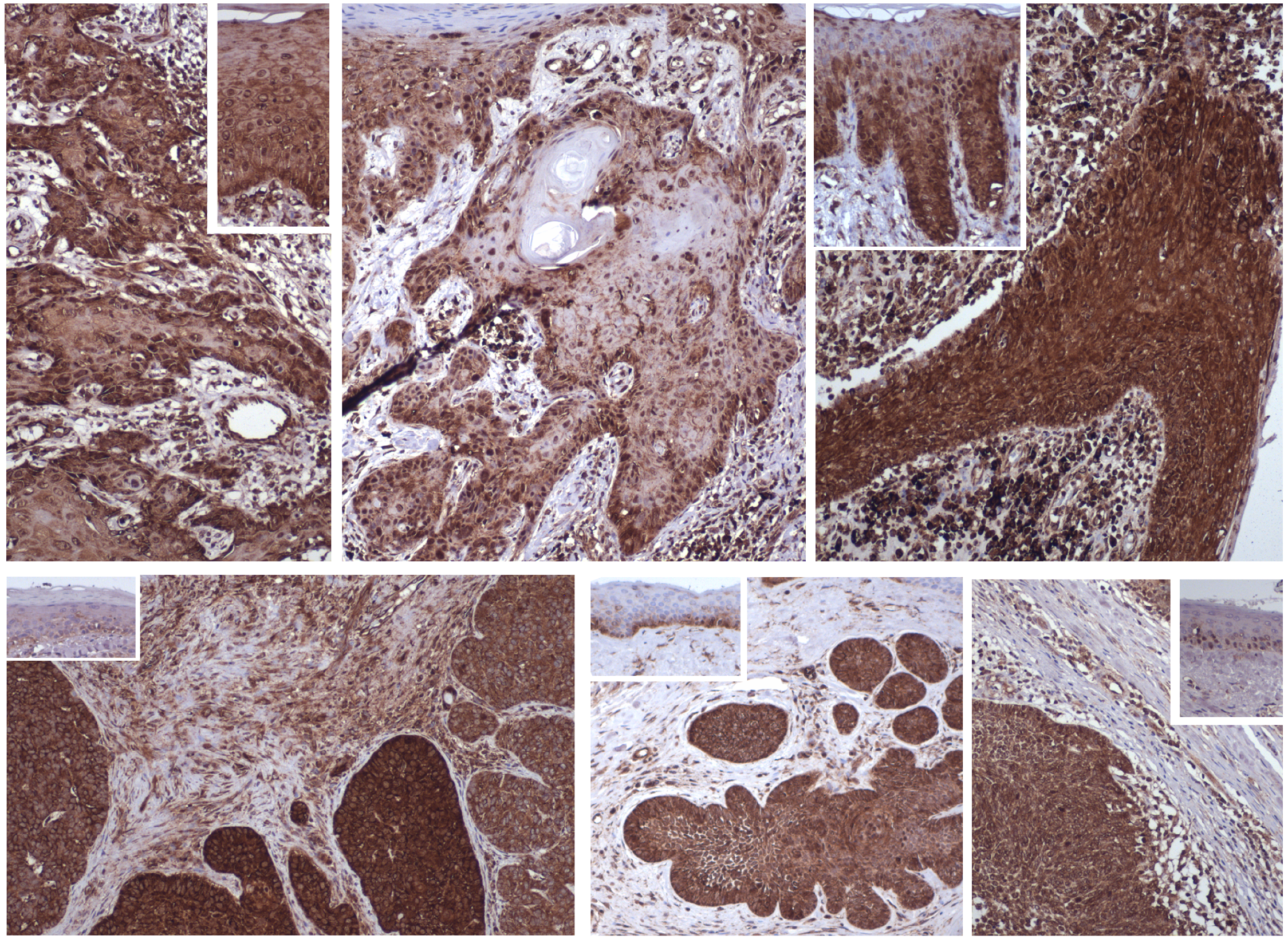

Supplement: Figure S5 — Expression of Wnt5a in SCC and BCC, as detected using an alternative antibody. Three SCC (top) and BCC (bottom) samples are shown, respectively. In each case, tumor – adjacent epidermis has been inserted to allow assessment of relative staining intensity. Wnt5a staining is strong and varies between homogenous (top right, bottom) and being stronger at tumor edge (top left and middle). Interstingly, the BCC sample on the bottom left suggest existence of tumor subclones with varying Wnt5a expression levels. Magnification 100×. (TIF) [file pone.0031827.s006.tif]

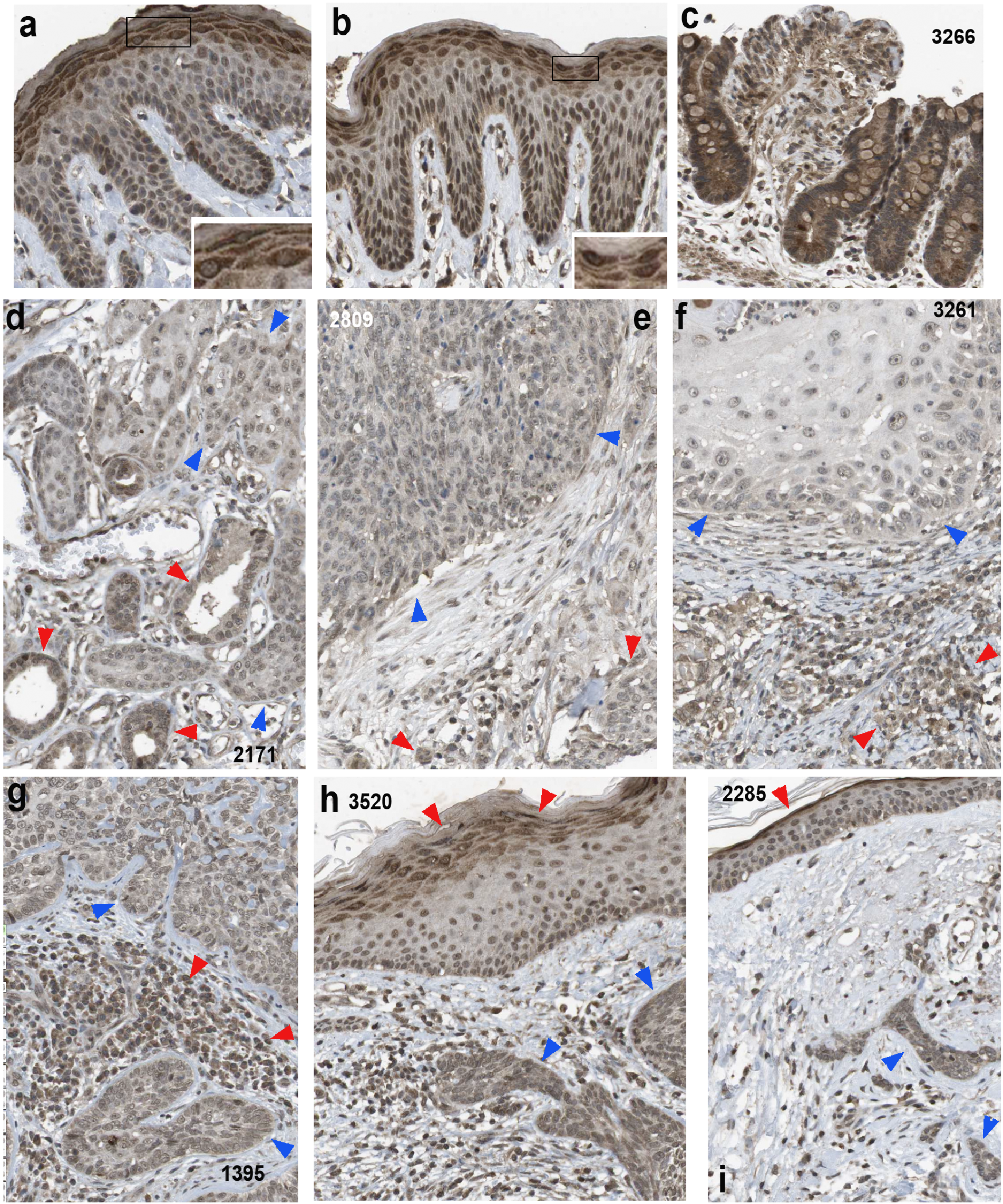

Supplement: Figure S6 — Immunohistochemistry of Axin2 in SCC and BCC. Samples were taken from the repository available at ProteinAtlas.org. Numbers in the tumor samples refer to patient IDs on the website. (a,b): normal anal/vulval skin samples at the website (normal epidermis was not available as separate samples. However, tumor-associated epidermis is shown in panels h,i) show strong expression in the granular layer, paralleling the location of activated β-catenin (see figs. 8,9). Note that despite intensive overall staining, the intracellular localisation of Axin2 appears to be perinuclear/cytoplasmic (insets in panels a,b). Panel C shows specific cytoplasmic staining in goblet cells of colonic mucosa as positive staining control. Further evidence for the validity of the staining results are the much stronger staining observed in colon carcinoma samples vs. normal colonic mucosa in numerous samples at the website, as well as stronger staining seen in ovarian cancer vs. normal ovarian tissue (in confirmation of the data published by Leung et al, “Activation of AXIN2 Expression by β-Catenin-T Cell Factor”, J Biol Chem 2002). Blue arrows: tumor tissue, red arrows: tumor-associated cells affording estimate of relative staining intensity in cluding eccrine glands (d), inflammatory stroma-infiltrate (e,f,g), as well as epidermis (h,i). (TIF) [file pone.0031827.s007.tif]
